# Supplementary material for: Structural and functional consequences of NEDD8 phosphorylation
Source: Nat Commun. 2021 Oct 12;12:5939. doi: 10.1038/s41467-021-26189-9 (PMC9020517; doi:10.1038/s41467-021-26189-9)
Supplement: Supplementary file 1 — Supplementary information [file 41467_2021_26189_MOESM1_ESM.pdf]

Structural and functional consequences of NEDD8 phosphorylation

Stuber et al.

Supplementary Information

SUPPLEMENTARY FIGURES

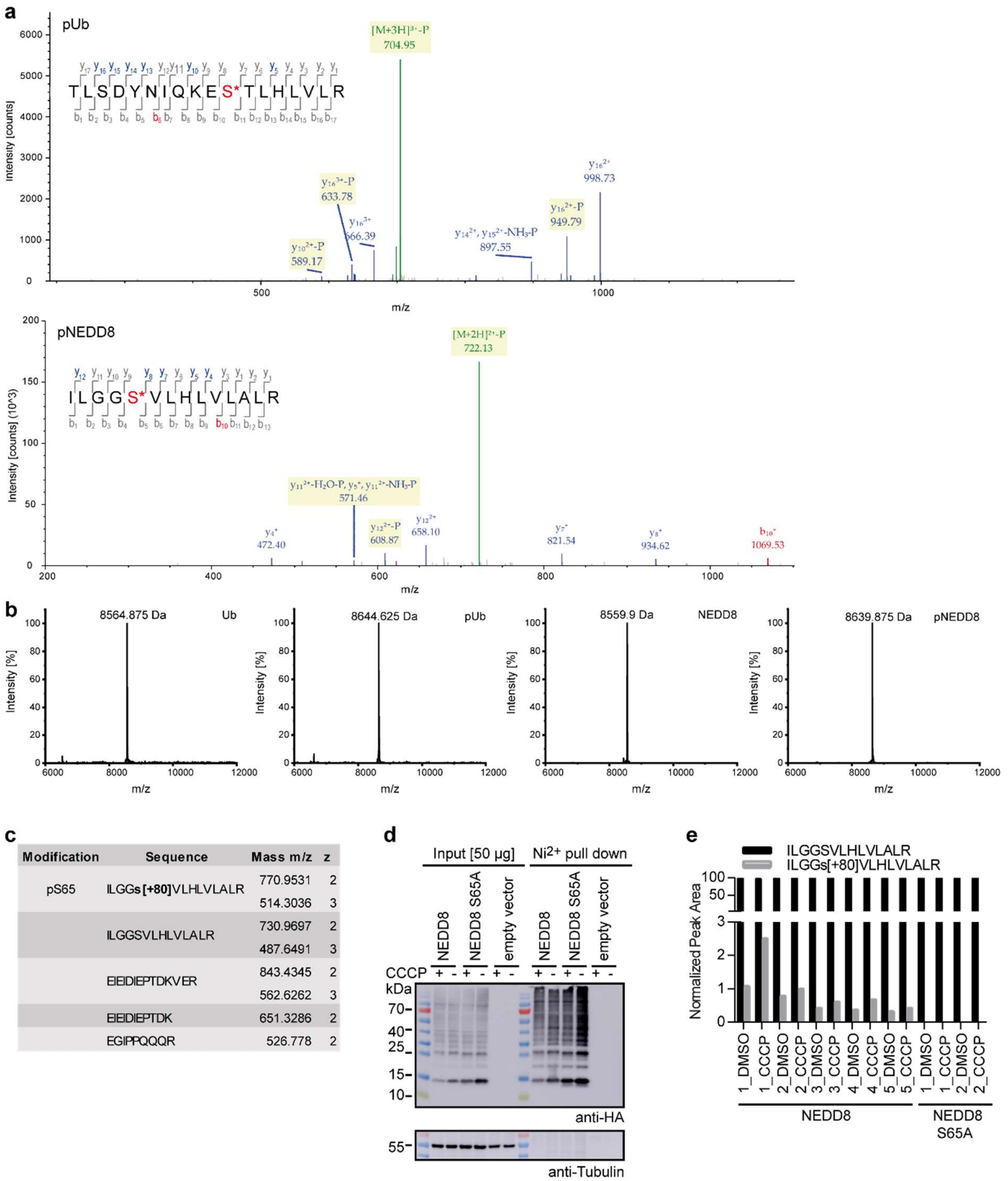

Modification	Sequence	Mass m/z	z
pS65	ILGGs[+80]VLHLVLALR	770.9531	2
		514.3036	3
	ILGGSVLHLVLALR	730.9697	2
		487.6491	3
	EIEIDIEPTDKVER	843.4345	2
		562.6262	3
	EIEIDIEPTDK	651.3286	2
	EGIPPQQQR	526.778	2

Supplementary Fig. 1. Mass spectrometric analysis of phosphorylated NEDD8. **a** LC-MS/MS analysis of PINK1-phosphorylated Ub and NEDD8 reveals phosphorylation at S65 (S* = Ser + 80 Da). **b** ESI-MS analysis demonstrates quantitative incorporation of pSer into Ub and NEDD8 via genetic code expansion (pUb: expected 8644.84 Da, observed 8644.625 Da; pNEDD8: expected 8639.97 Da, observed 8639.875 Da. **c** NEDD8 peptide list used for targeted proteomics to identify pNEDD8 levels. **d** Upon affinity enrichment, 20% of the HA-His-NEDD8 elution fraction were analyzed by SDS-PAGE followed by Western blot analysis. The Western blot shown is representative of three independent experiments. **e** Normalized peak areas of the peptides indicated for the biological replicates. Source data are provided as a Source Data file.

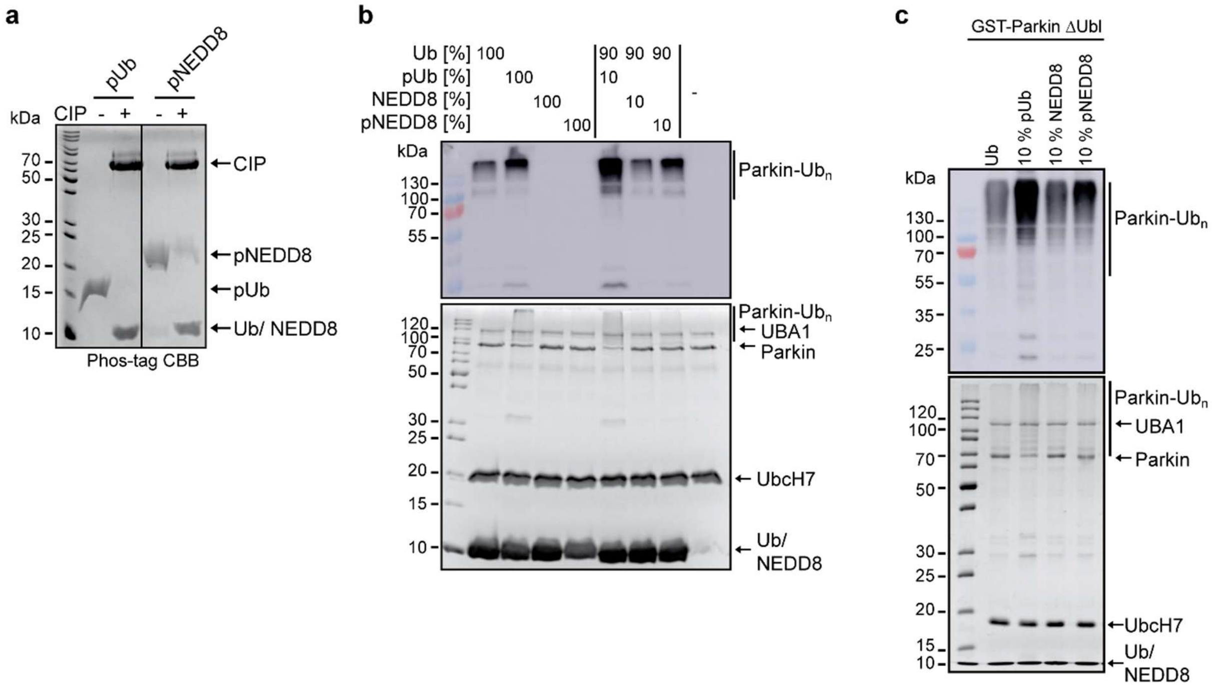

Supplementary Fig. 2. Biochemical analysis of phosphorylated NEDD8. a pUb and pNEDD8 were incubated in the absence (-) or presence (+) of alkaline phosphatase (CIP). Upon treatment, reactions products were analyzed by Phos-tag SDS-PAGE followed by Coomassie blue staining. **b/c** Parkin autoubiquitylation was performed with Parkin or Parkin ΔUbl as described in Methods in the presence of the Ub/NEDD8 variants indicated. Reactions were stopped after 60 min and analyzed by SDS-PAGE followed by Western blot analysis with an anti-Ub antibody (upper panels) or by Coomassie blue staining (lower panels). Running positions of molecular mass markers, unmodified Parkin, ubiquitylated forms of Parkin, UBA1, UbcH7, and Ub/NEDD8 variants are indicated. The results shown are representative of three independent experiments. Source data are provided as a Source Data file.

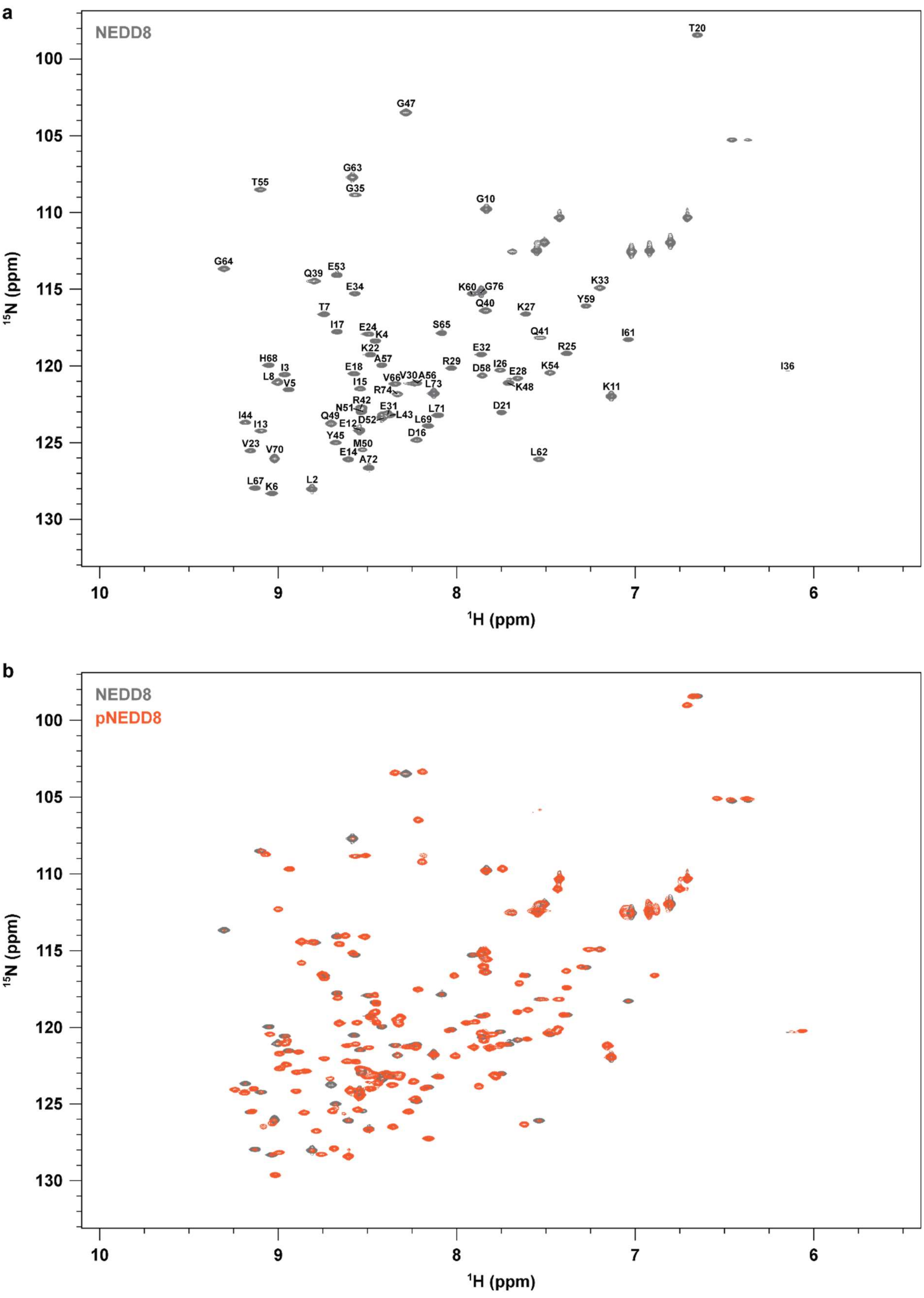

Supplementary Fig. 3. NMR spectra of unmodified NEDD8 and phosphorylated NEDD8 (pNEDD8).
a Two-dimensional ^{1}H-^{15}N HSQC NMR spectrum of NEDD8 including labels for the assignment of backbone amide resonance signals by using the one letter code for amino acids followed by the position in the primary sequence. **b** Two-dimensional ^{1}H-^{15}N HSQC NMR spectra of NEDD8 colored in anthracite and of pNEDD8 colored in orange are superimposed to identify resonances corresponding to traces of unmodified NEDD8 in the pNEDD8 preparation (cf. Fig. 2a). As outlined in Methods, upon expression in bacteria pNEDD8 samples contain unmodified NEDD8 that could not be separated from pNEDD8.

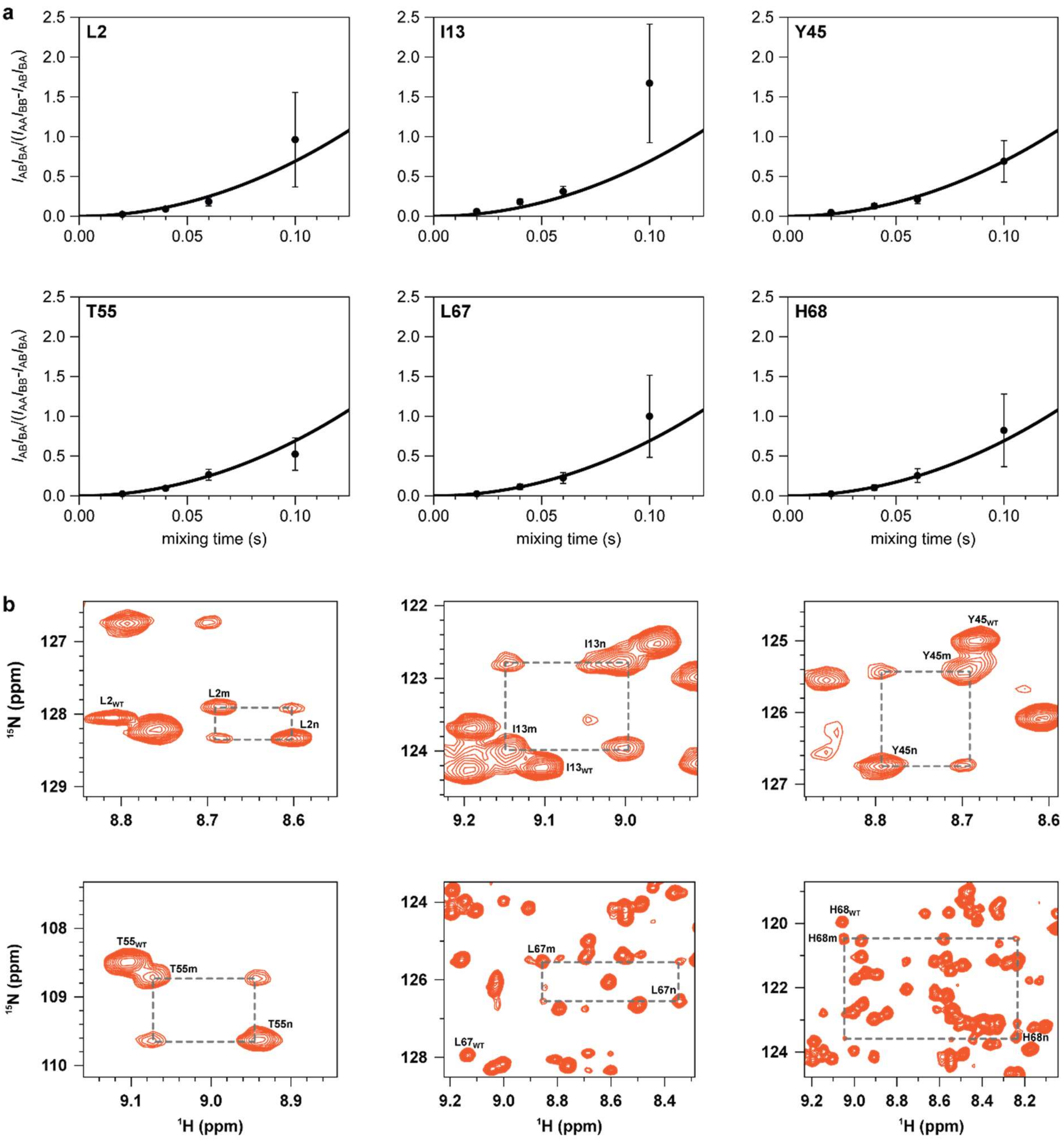

Supplementary Fig. 4. ZZ exchange NMR spectroscopy of pNEDD8. a Build-up curves resulting from a global fitting procedure on the composite ratios of signal heights of auto-peaks and cross-peaks according to equation (3) listed in Methods are shown for six selected residues of pNEDD8. The experiment with a mixing time of 40 ms was performed twice to yield the overall standard deviation from all auto- and cross-peak intensities of the six selected residues and the standard deviation was propagated when calculating the composite signal height ratios resulting in the error bars depicted. **b** Excerpts from the ZZ exchange spectrum acquired using a mixing time of 40 ms comprising the resonance signals of corresponding residues from (a) illustrate the interconversion between the relaxed state (m) and the retracted state (n) of pNEDD8. The connectivity of auto-peaks and cross-peaks belonging to the same residue are indicated by dashed lines. Corresponding amide proton resonance signals originating from unmodified NEDD8 present in the sample (see Fig. S3) are indicated by WT. Source data are provided as a Source Data file.

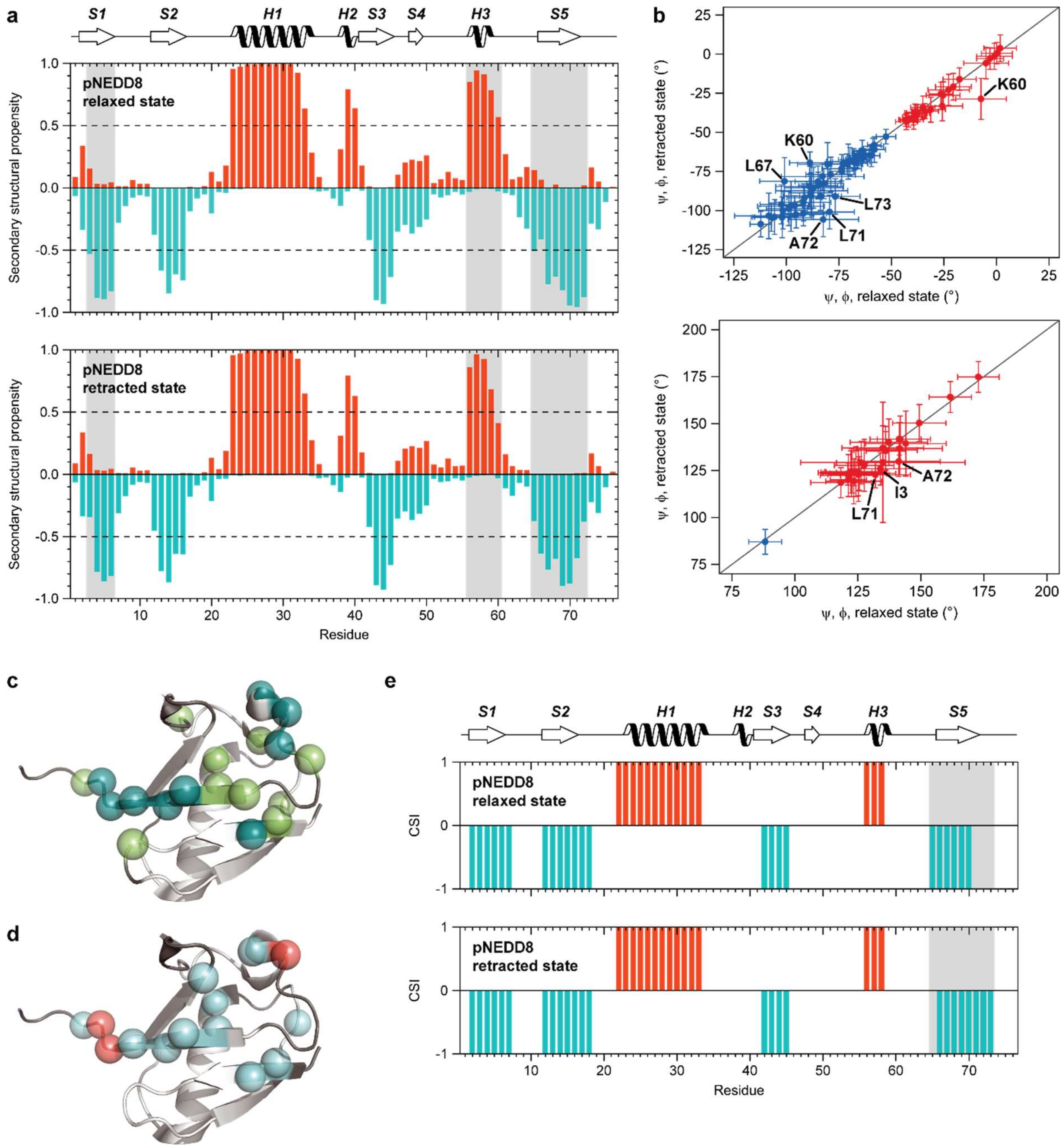

Supplementary Fig. 5. Secondary structure analysis of pNEDD8 by TALOS-N and use of chemical shift indices. a The secondary structural propensities are represented as a bar graph for the relaxed state (upper panel) and the retracted state conformation (lower panel) of pNEDD8. Positive values colored in red indicate helices whereas negative values colored in cyan indicate β-strands. In order to highlight the structural differences of I3, K60, V66 and A72, the corresponding secondary structural elements are shaded in grey in the background. The secondary structural elements observed in the crystal structure of unmodified NEDD8 (PDB ID 1NDD) are depicted on top for reference. **b** The torsion angles ψ and φ of the relaxed and retracted state conformation of pNEDD8 obtained from the TALOS analysis are compared in a correlation plot. The dihedrals are colored in blue (ψ) and red (φ), and the residues with the highest deviations are additionally labeled. The error bars indicate the standard deviation of the torsion angles calculated on basis of the best database matches of heptapeptides in terms of chemical shifts and residue type as identified by TALOS-N. The number of heptapetides included is either 25 or 10 depending on the clustering in the Ramachadran plot. **c** Weighted chemical shift perturbations (CSP, $\Delta\omega$) from Fig. 2d comparing the relaxed state and the retracted state of pNEDD8 are highlighted on the NMR solution structure of unmodified NEDD8 (PDB ID 2KO3) in cartoon mode. The Cα atoms of residues possessing significant CSP values are additionally shown as spheres.

Residues with CSP values higher than the mean are colored in green and residues with CSP values higher than the mean plus one standard deviation are colored in teal. **d** Differences in torsion angles (ψ, φ) between the relaxed state and the retracted state of pNEDD8 obtained by TALOS-N and as illustrated in Fig. S5b are highlighted on the unmodified NEDD8 structure as in c. Residues with either ψ or φ angles deviating more than 5° between both states are colored in cyan, whereas residues with both dihedral angles deviating more than 5° are colored in red. Residues without available structural information are colored in dark gray in (c) and (d). **e**, Chemical shift indices (CSI) for the relaxed state (upper panel) and the retracted state conformation (lower panel) of pNEDD8 were calculated by the CSI 3.0 web server. Bars from 0 to 1 colored in red indicate helices and bars from 0 to -1 colored in cyan indicate β-strands. The region around the C-terminal β_5-strand is shaded in grey to highlight the retraction of the corresponding strand in the retracted state in comparison to the relaxed state. Note that in addition, CSI analysis revealed an extension of the β_5-strand by two residues (from S65-V70 to V66-L73). The secondary structural elements of the crystal structure of unmodified NEDD8 (PDB ID 1NDD) are depicted on top for reference.

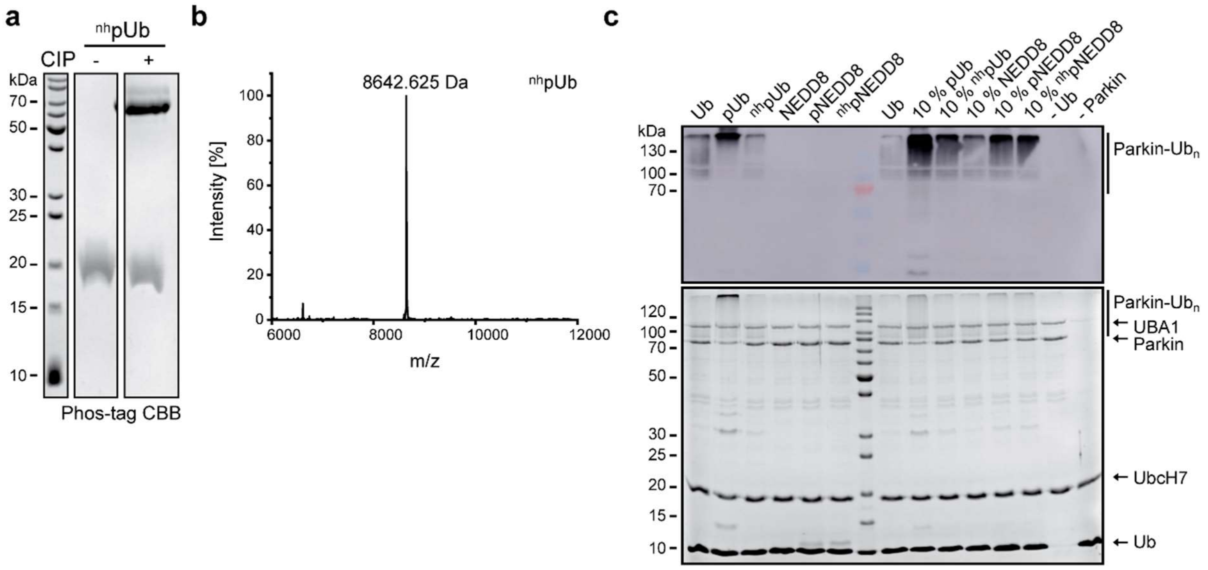

Supplementary Fig. 6. Analysis of ubiquitin containing a non-hydrolyzable phosphoserine analog (^{nh}pUb). a ^{nh}pUb was incubated in the absence (-) or presence (+) of alkaline phosphatase (CIP). Upon treatment, reaction products were analyzed by Phos-tag SDS-PAGE followed by Coomassie blue staining. The experiment shown is representative of three independent experiments. **b** ESI-MS analysis demonstrates the incorporation of ^{nh}pSer into Ub (^{nh}pUb: expected 8642.84 Da, observed 8642.625 Da). **c** Parkin autoubiquitylation was performed as described in Methods in the presence or absence of the Ub/NEDD8 variants indicated. Reactions were stopped after 60 min and analyzed by SDS-PAGE followed by Western blot analysis with an anti-Ub antibody (upper panels) or by Coomassie blue staining (lower panels). Running positions of molecular mass markers, unmodified Parkin, ubiquitylated forms of Parkin, UBA1, UbcH7, and Ub variants are indicated. The experiment shown is representative of three independent experiments. Source data are provided as a Source Data file.

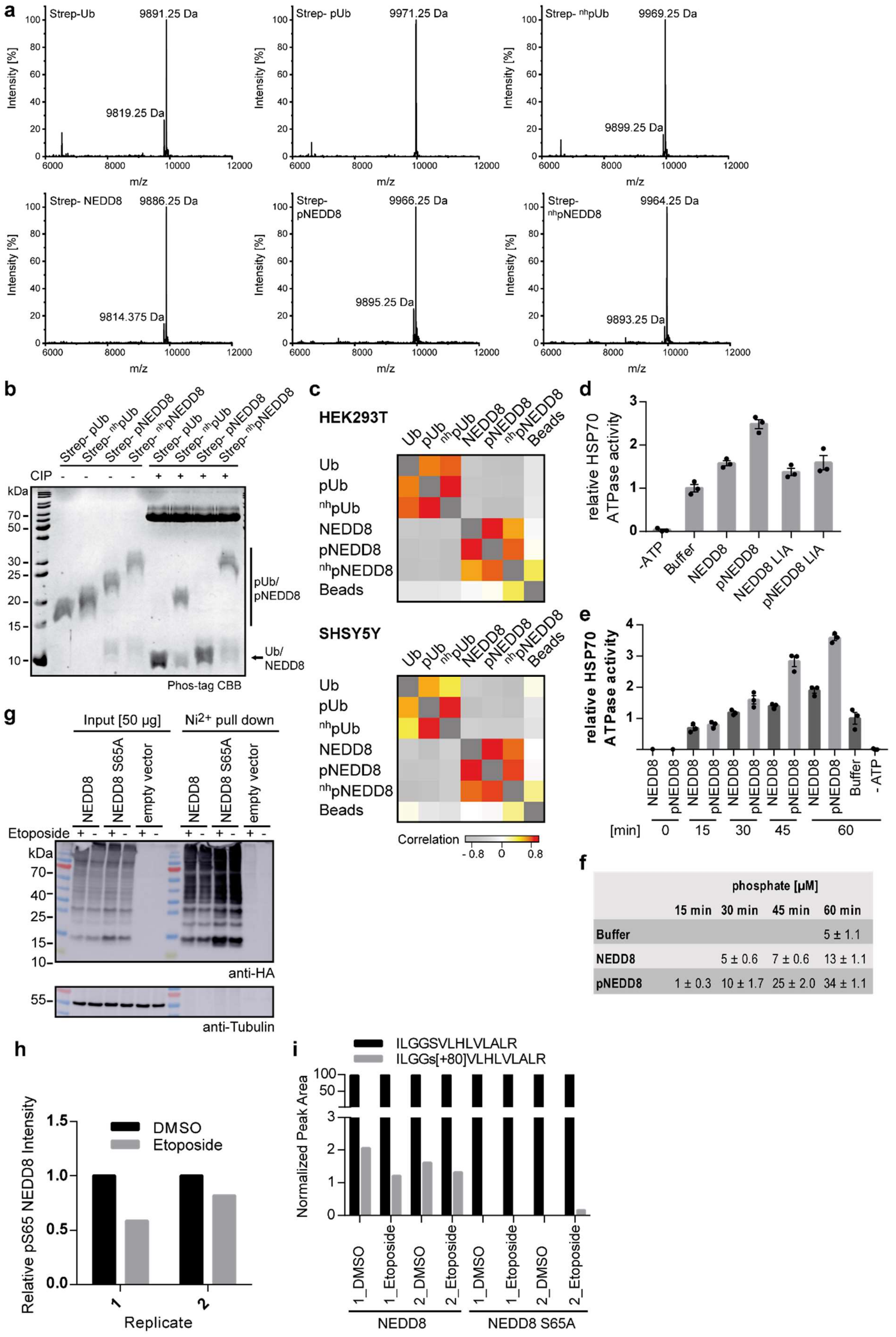

	phosphate [µM]			
	15 min	30 min	45 min	60 min
Buffer				5 ± 1.1
NEDD8		5 ± 0.6	7 ± 0.6	13 ± 1.1
pNEDD8	1 ± 0.3	10 ± 1.7	25 ± 2.0	34 ± 1.1

Supplementary Fig. 7. Analysis of Strep-tagged Ub and NEDD8 variants. a ESI-MS analysis confirms the incorporation of pSer or ^{nh}pSer into Ub and NEDD8 (Strep-Ub: expected 9891.28 Da, observed 9891.25 Da; Strep-pUb: expected 9971.28 Da, observed 9971.25 Da; Strep-^{nh}pUb: expected 9969.28 Da, observed 9969.25 Da; Strep-NEDD8: expected 9886.4 Da, observed 9886.25 Da; Strep-pNEDD8: expected 9966.4 Da, observed 9966.25 Da; Strep-^{nh}pNEDD8: expected 9964.4 Da, observed 9964.25 Da; the smaller masses indicated correspond to the N-terminal truncation of an alanine in front of the Strep-tag II: calculated: -71.08 Da). **b** N-terminal Strep-tagged Ub and NEDD8 variants were incubated in the absence (-) or presence (+) of alkaline phosphatase (CIP). Upon treatment, reactions products were analyzed by Phos-tag SDS-PAGE followed by Coomassie blue staining. Unlike pUb, pNEDD8 samples contain traces of unmodified NEDD8 resulting from differences in the purification scheme (see Methods). The experiment shown is representative of three independent experiments. **c** Heatmaps of pairwise Pearson correlation between significantly enriched interactors of the different bait molecules identified with HEK293T cell extracts and SHSY5Y cell extracts. Strong correlation is indicated in red, medium correlation in yellow, no correlation in white and anti-correlation in grey. Dark grey indicates no correlation value calculated. **d** The relative stimulation of HSP70 ATPase activity was measured in presence of NEDD8, pNEDD8, NEDD8 LIA and pNEDD8 LIA and compared to HSP70 basal ATPase activity (n=3±SEM independent experiments). **e** The relative stimulation of HSP70 ATPase activity was measured in presence of NEDD8 or pNEDD8 at the times indicated and compared to the basal ATPase activity of HSP70 after 60 min (n=3±SEM independent experiments). **f** The results obtained in the experiment shown in **e** was used to calculate absolute values of released phosphate using a phosphate standard. **g-i**, Phosphorylation of S65 of NEDD8 under DNA double-strand break stress. **g** 20% of the HA-His-NEDD8 Ni^{2+} pull down elution fraction were analyzed by SDS-PAGE followed by Western blot analysis. **h** 80 % of the HA-His-NEDD8 Ni^{2+} pull down elution fraction were tryptic digested, subjected to targeted LC-MS/MS and the normalized relative intensity of the tryptic S65 NEDD8 peptide compared between conditions. Shown are two biological replicates measured in technical duplicates. **i** Normalized peak areas of the peptides indicated for two biological replicates. Source data are provided as a Source Data file.

SUPPLEMENTARY TABLES

Supplementary Table 1. Oligonucleotides used for cloning.

Oligonucleotide	Sequence
NdeI pGEX_F	CAGGAAACAGTACATATGTCCCCTATACTAGG
NdeI pGEX_R	CCT AGT ATA GGG GAC ATA TGT ACT GTT TCC TG
pKS_F	CCGACATCATAACGGTTCTG
Ub S65TAG_F	CAACATTCAGAAAGAATAGACGCTTCACTTGG
Ub S65TAG_R	CCA AGT GAA GCG TCT ATT CTT TCT GAA TGT TG
NdeI NEDD8_F	GACTGTCATATGCTAATTAAAGTGAAGACGC
BamHI NEDD8_R	GAG CTC GGA TCC CGT TGT ACT CCT CCT CTA AGA GCC AA
BamHI Parkin_F	GATCATGGATCCATGATAGTGTTTGTCAGG
EcoRI Parkin_R	GATCATGAATTCCTACACGTCGAACCAG
Nedd8 S65TAG_F	TTTAGGTGGTTAGGTCCTTCACCTGGTGTT
NEDD8 S65TAG_R	AAC ACC AGG TGA AGG ACC TAA CCA CCT AAA
Strep-Ub_F	gcagttcgaaaagggtgcaATGCAAATCTTCGTCAAAAC
Strep-Ub_R	gggtggctccagcttgccatATGTACTGTTTCCTGTGTG
Strep-Nedd8_F	gcagttcgaaaagggtgcaATGCTAATTAAAGTGAAGAC
Strep-Nedd8_R	gggtggctccagcttgccatATGTACTGTTTCCTGTGTG
NdeI HSPA8_F	GATCATCATATGTCCAAGGGACCTGC
BamHI HSPA8_R	GATCACGGATCCTTAACCAAGGGAAAGAGGAG
Parkin H302_F	CCAACTCCTTGATTAAAGAGCTCGCGCACTTC
Parkin H302_R	GAA GTG CGC GAG CTC TTT AAT CAA GGA GTT GG
Parkin ΔUbl_F	GGTCAAGAAATGAATGCAAC
Parkin ΔUbl_R	GGATCCAACAGATGCACG
NEDD8 deltaGG_F	CACCTGGTGTTGGCTCTTAGATAATGATAACTC
NEDD8 deltaGG_R	GAG TTA TCA TTA TCT AAG AGC CAA CAC CAG GTG
NEDD8 L8A_F	TAATTAAAGTGAAGACGGCGACCGGAAAGG
NEDD8 L8A_R	CCTTTCCGGTCGCCGTCTTCACTTTAATTA
NEDD8 I44A_F	AGAGGCTCGCCTACAGTGGCAAGCAGATGA
NEDD8 I44A_R	TCATCTGCTTGCCACTGTAGGCGAGCCTCT
