# Supplementary material for: Structural and functional consequences of NEDD8 phosphorylation
Source: Nat Commun. 2021 Oct 12;12:5939. doi: 10.1038/s41467-021-26189-9 (PMC9020517; doi:10.1038/s41467-021-26189-9)
Supplement: Supplementary file 3 — Reporting Summary [file 41467_2021_26189_MOESM3_ESM.pdf]

nature research

Corresponding author(s): Michael Kovermann, Andreas Marx & Martin Scheffner

Last updated by author(s): Sep 17, 2021

Reporting Summary

Nature Research wishes to improve the reproducibility of the work that we publish. This form provides structure for consistency and transparency in reporting. For further information on Nature Research policies, see our Editorial Policies and the Editorial Policy Checklist.

Statistics

For all statistical analyses, confirm that the following items are present in the figure legend, table legend, main text, or Methods section.

n/a	Confirmed	
☐	☒	The exact sample size (n) for each experimental group/condition, given as a discrete number and unit of measurement
☐	☒	A statement on whether measurements were taken from distinct samples or whether the same sample was measured repeatedly
☐	☒	The statistical test(s) used AND whether they are one- or two-sided<br>*Only common tests should be described solely by name; describe more complex techniques in the Methods section.*
☒	☐	A description of all covariates tested
☐	☒	A description of any assumptions or corrections, such as tests of normality and adjustment for multiple comparisons
☐	☒	A full description of the statistical parameters including central tendency (e.g. means) or other basic estimates (e.g. regression coefficient) AND variation (e.g. standard deviation) or associated estimates of uncertainty (e.g. confidence intervals)
☐	☒	For null hypothesis testing, the test statistic (e.g. F, t, r) with confidence intervals, effect sizes, degrees of freedom and P value noted<br>*Give P values as exact values whenever suitable.*
☒	☐	For Bayesian analysis, information on the choice of priors and Markov chain Monte Carlo settings
☒	☐	For hierarchical and complex designs, identification of the appropriate level for tests and full reporting of outcomes
☐	☒	Estimates of effect sizes (e.g. Cohen's d, Pearson's r), indicating how they were calculated

Our web collection on statistics for biologists contains articles on many of the points above.

Software and code

Policy information about availability of computer code

Data collection	All MS/MS data was collected on an QExcactive HF Hybrid Quadrupole-Orbitrap operated with Tune (version 2.9) or on a LTQ Oribtrap XL (Thermo). MS data were collected on a micrOTOF II (Bruker). All NMR experiments were performed at T = 298 K on a Bruker Avance NEO 800 MHz spectrometer equipped with cryogenic triple resonance TCI or quadruple resonance QCI probes. Absorbance was measured on an TECAN infinite F500 plate reader. Gel and Western Blot pictures were taken on an Amersham imager 600.
Data analysis	MaxQuant (version 1.6.8), Perseus software (version 1.6.10.50), Proteome Discoverer 1.4 , Mascot 2.6, Compass DataAnalysis 4.1 (Bruker), NMRPipe (version 10.1), NMRViewJ (version 8.0.a27), TALOS-N (version 4.21), CSI 3.0, GraphPad Prism 6 (GraphPad Software), Amersham Imager 600 Analysis Software (GE)

For manuscripts utilizing custom algorithms or software that are central to the research but not yet described in published literature, software must be made available to editors and reviewers. We strongly encourage code deposition in a community repository (e.g. GitHub). See the Nature Research guidelines for submitting code & software for further information.

Data

Policy information about availability of data

All manuscripts must include a data availability statement. This statement should provide the following information, where applicable:

- Accession codes, unique identifiers, or web links for publicly available datasets
- A list of figures that have associated raw data
- A description of any restrictions on data availability

NMR resonance assignments have been deposited in the Biological Magnetic Resonance Data Bank with the following accession numbers: 50466 [https://bmrb.io/data_library/summary/index.php?bmrbId=50466] (NEDD8), 50467 [https://bmrb.io/data_library/summary/index.php?bmrbId=50467] (pNEDD8 relaxed state), and 50468 [https://bmrb.io/data_library/summary/index.php?bmrbId=50468] (pNEDD8 retracted state).

Preexisting NMR resonance assignments used in this study are available under the accession codes 10062 [https://bmrb.io/data_library/summary/index.php?bmrbId=10062] (His-tagged NEDD8), 36082 [https://bmrb.io/data_library/summary/index.php?bmrbId=36082] (pUb relaxed state), and 36081 [https://bmrb.io/data_library/summary/index.php?bmrbId=36081] (pUb retracted state).
The NEDD8 crystal structure data, the NMR solution structure of NEDD8, and the NMR solution structure of ubiquitin used in this study are available in the Protein Data Bank under the accession codes 1NDD [https://www.rcsb.org/structure/1NDD], 2KO3 [https://www.rcsb.org/structure/2KO3], and 1D3Z [https://www.rcsb.org/structure/1D3Z], respectively.
The mass spectrometry proteomics data have been deposited to the ProteomeXchange Consortium (http://proteomecentral.proteomexchange.org) via the PRIDE partner repository67 with the dataset identifier PXD021143 [https://www.ebi.ac.uk/pride/archive/projects/PXD021143] for the affinity enrichment data and the dataset identifier PXD027477 [https://www.ebi.ac.uk/pride/archive/projects/PXD027477] for the pNEDD8 analysis data.
Figures with associated raw data: Figures 1-6 and Supplementary Figures 1-7

Field-specific reporting

Please select the one below that is the best fit for your research. If you are not sure, read the appropriate sections before making your selection.

[x] Life sciences [ ] Behavioural & social sciences [ ] Ecological, evolutionary & environmental sciences

For a reference copy of the document with all sections, see nature.com/documents/nr-reporting-summary-flat.pdf

Life sciences study design

All studies must disclose on these points even when the disclosure is negative.

Sample size	Sample sizes were not predetermined based on statistical methods, but were chosen according to the standards of the field (at least three independent biological replicates for each condition). AE-MS samples were prepared in biological triplicates for all investigated samples, and each of these was measured with technical duplicates.<br>In cellulo NEDD8 phosphorylation MS samples were prepared in biological quintuplicates and measured in technical duplicates for replicate 1&2 and in triplicates for replicate 3-5 for the CCCP treatment. For the etoposide treatment the MS samples were prepared in biological duplicates and each of these was measured with technical duplicates.<br>HSP70 ATPase activity measurements were analyzed in triplicates.
Data exclusions	No data was excluded, only search criteria applied as described in the mansucript.
Replication	AE-MS samples were prepared in biological triplicates for all investigated samples, and each of these was measured with technical duplicates.<br>In cellulo NEDD8 phosphorylation MS samples were prepared in biological quintuplicates and measured in technical duplicates for replicate 1&2 and in triplicates for replicate 3-5 for the CCCP treatment. For the etoposide treatment the MS samples were prepared in biological duplicates and each of these was measured with technical duplicates.<br>In vitro ubiquitylation and phosphorylation experiments were performed at least three times.<br>HSP70 ATPase activity measurements were analyzed in triplicates.<br>All attempts at replication were successful.
Randomization	n/a, No human or animal subjects were used in the study. Randomization is generally not used in the field and not applicable for the approaches used.
Blinding	n/a, Investigators and samples were not blinded. Blinding is not used in the field.

Reporting for specific materials, systems and methods

We require information from authors about some types of materials, experimental systems and methods used in many studies. Here, indicate whether each material, system or method listed is relevant to your study. If you are not sure if a list item applies to your research, read the appropriate section before selecting a response.

Materials & experimental systems

n/a	Involved in the study	
[ ]	[x]	Antibodies
[ ]	[x]	Eukaryotic cell lines
[x]	[ ]	Palaeontology and archaeology
[x]	[ ]	Animals and other organisms
[x]	[ ]	Human research participants
[x]	[ ]	Clinical data
[x]	[ ]	Dual use research of concern

Methods

n/a	Involved in the study	
[x]	[ ]	ChIP-seq
[x]	[ ]	Flow cytometry
[x]	[ ]	MRI-based neuroimaging

Antibodies

Antibodies used	Primary antibody: anti-Ub antibody FK2 (ST1200, Merck), anti- HDAC6 (7612S, Cell Signaling Technologies), anti-USP16 (HPA021140, Sigma), anti-UBA1 (4891S, Cell Signaling Technologies) anti-UBE2M (4913S, Cell Signaling Technologies), anti-HSPA8 (PA5-27337, Thermo Fisher), anti-HA (901533, Biolegend), anti-Tubulin (ab7291, Abcam), anti-Parkin PRK8 (sc-32282, Santa Cruz) Secondary antibody: mouse (115-035-062, Jackson ImmunoResearch), rabbit (111-035-003, Jackson ImmunoResearch)
Validation	Validation of each primary antibody is available on the manufacturers website under the hyperlinks provided below.<br>anti-Ub antibody FK2 (ST1200, Merck), [https://www.merckmillipore.com/DE/de/product/Anti-Ubiquitin-Mouse-mAb-FK2,EMD_BIO-ST1200]<br>anti- HDAC6 (7612S, Cell Signaling Technologies), [https://www.cellsignal.com/products/primary-antibodies/hdac6-d21b10-rabbit-mab/7612]<br>anti-USP16 (HPA021140, Sigma), [https://www.sigmaaldrich.com/DE/de/product/sigma/hpa021140]<br>anti-UBA1 (4891S, Cell Signaling Technologies), [https://www.cellsignal.com/products/primary-antibodies/ube1a-b-antibody/4891]<br>anti-UBE2M (4913S, Cell Signaling Technologies), [https://www.cellsignal.com/products/primary-antibodies/ubc12-antibody/4913]<br>anti-HSPA8 (PA5-27337, Thermo Fisher), [https://www.thermofisher.com/antibody/product/HSC70-Antibody-Polyclonal/PA5-27337]<br>anti-HA (901533, Biolegend), [https://www.biolegend.com/en-ie/products/purified-anti-ha-11-epitope-tag-antibody-11374]<br>anti-Tubulin (ab7291, Abcam), [https://www.abcam.com/alpha-tubulin-antibody-dm1a-loading-control-ab7291.html]<br>anti-Parkin PRK8 (sc-32282, Santa Cruz), [https://www.scbt.com/de/p/parkin-antibody-prk8]

Eukaryotic cell lines

Policy information about cell lines

Cell line source(s)	ATCC (HEK 293T, SHSY5Y)
Authentication	The cell lines used were not authenticated.
Mycoplasma contamination	Cell line were not tested for Mycoplasma contamination.
Commonly misidentified lines (See ICLAC register)	No commonly misidentified cell lines were used in this study.
